# The Effectiveness of Physical Exercise on Bone Density in Osteoporotic Patients

**DOI:** 10.1155/2018/4840531

**Published:** 2018-12-23

**Authors:** Maria Grazia Benedetti, Giulia Furlini, Alessandro Zati, Giulia Letizia Mauro

**Affiliations:** ^1^Physical Medicine and Rehabilitation Unit, IRCCS-Istituto Ortopedico Rizzoli, Bologna, Italy; ^2^Rehabilitation Unit, Paolo Giaccone Hospital, Palermo, Italy

## Abstract

Physical exercise is considered an effective means to stimulate bone osteogenesis in osteoporotic patients. The authors reviewed the current literature to define the most appropriate features of exercise for increasing bone density in osteoporotic patients. Two types emerged: (1)* weight-bearing aerobic exercises*, i.e., walking, stair climbing, jogging, and Tai Chi. Walking alone did not appear to improve bone mass; however it is able to limit its progressive loss. In fact, in order for the weight-bearing exercises to be effective, they must reach the mechanical intensity useful to determine an important ground reaction force. (2)* Strength and resistance exercises*: these are carried out with loading (lifting weights) or without (swimming, cycling). For this type of exercise to be effective a joint reaction force superior to common daily activity with sensitive muscle strengthening must be determined. These exercises appear extremely site-specific, able to increase muscle mass and BMD only in the stimulated body regions. Other suggested protocols are multicomponent exercises and whole body vibration.* Multicomponent exercises* consist of a combination of different methods (aerobics, strengthening, progressive resistance, balancing, and dancing) aimed at increasing or preserving bone mass. These exercises seem particularly indicated in deteriorating elderly patients, often not able to perform exercises of pure reinforcement. However, for these protocols to be effective they must always contain a proportion of strengthening and resistance exercises. Given the variability of the protocols and outcome measures, the results of these methods are difficult to quantify. Training with* whole body vibration (WBV):* these exercises are performed with dedicated devices, and while it seems they have effect on enhancing muscle strength, controversial findings on improvement of BMD were reported. WBV seems to provide good results, especially in improving balance and reducing the risk of falling; in this, WBV appears more efficient than simply walking. Nevertheless, contraindications typical of senility should be taken into account.

## 1. Introduction

According to the literature, the level of bone loss in a postmenopausal woman increases with age, respectively, with a loss of 0.6%, 1.1%, and 2.1% per year for the 60-69, 70-79, and> 80 age groups [1]. More precisely, the loss is 1.5% per year for the spine and 1.1% - 1.4% for the femoral neck in the first 4-5 years [2]. In the following years, the loss is less rapid as it is the immediate postmenopausal period characterized by a greater speed and entity of bone loss [3]. Although exercise is widely recommended as one of the primary preventive strategies to reduce the risk of osteoporosis [3], its effects on bone are controversial. In fact, not all types of exercise have the same positive effect on bone mineral density (BMD). While there is evidence that exercise induces an increase in bone mass in younger subjects, this effect in adults and elderly people remains questionable [4]. In elderly people, the results of the studies indicate that exercise may increase the thickness and resistance of cortical bone at loaded skeletal sites [5]. However, it seems that the improvement on bone strength induced by exercise in older adults is likely to be due to a lower loss of endocortical bone and/or an increase in tissue density, rather than an increase in bone size (periosteal apposition), typical of young subjects. Supposing that trabecular bone architecture can adapt to increased loading, the effects of physical activity on thickness, number, separation, and orientation of trabecular elements in human bones are not known due to the limited resolution of most current commonly used imaging techniques [5].

Therefore, there is considerable interest in defining the adequate dosage and characteristics of exercises to improve bone strength in osteoporosis, in order to develop appropriate guidelines, given the fact that economic and social costs appear to be in a progressive and constant growth in relation to the aging of the population [6, 7].

In the past years, many studies [1, 8–12] have reported very consistent results on the beneficial effects of exercise on BMD of the lumbar spine and the femur in menopausal women and, in general, in old age. Various exercises have been described to stimulate bone growth and preserve the bone mass; the optimal interventions are those favoring a mechanical stimulus on bone both through antigravity loading and the stress exerted on muscles [8–10]. In general, therapeutic exercises for osteoporosis can be ranked in two types of activities:

- *Weight-bearing aerobic exercises*, such as impact activities or any other exercise in which arms, feet, and legs are bearing the weight, (i.e., walking, stair climbing, jogging, volleyball, tennis and similar sports, Tai Chi, and dancing).
- *Strength end/or resistance exercises*, in which the joints are moved against some kind of resistance, in the form of free weights, machines, tubing, or one's own body weight [9].

However, it is still not clear which exercise is the best suited and how long it would take to obtain an appropriate result. For example, the SIOMMS guidelines [13] recommend performing a minimum of physical activity, such as walking, for 30 minutes every day, despite the lack of available evidence. Meanwhile the position paper in the American College of Sports Medicine [5] suggests, during adulthood, to carry out weight-bearing endurance activities (i.e., tennis, stair climbing, and jogging), activities that involve jumping (volleyball, basketball), and resistance exercise (weight lifting) with moderate or high intensity, 3-5 times a week for 30-60 minutes, possibly in combination.

The effects of exercise on bone tissue have gained an important contribution also from studies on sport athletes. Numerous publications have linked physical exercise, bone metabolism markers, and bone mineral density [36]. While the isolated exercise (single bout training) seems to give a fleeting osteogenetic stimulation, a longer training, for example, 2 times a day for 5 weeks, seems to provide a better stimulation. Furthermore, aerobic exercise seems to be particularly effective in the enzymatic activation of the osteoblasts. These observations underscore the importance of combining aerobic and anaerobic exercises in osteogenetic protocols. Furthermore, after a longer period of training (6-12 months), the sedentary and untrained individuals obtained better results in BMD than the already trained individuals with significant osteogenetic activity without increasing the reabsorption indexes. Probably individuals already trained with the continuation of exercise only maintained the good bone metabolic level already reached, which can not indefinitely increase. Regarding the type of exercise, sport shows us clearly how the activities performed in weight bearing, including high impact and endurance mechanical components, are more effective in increasing the BMD of limited or nonimpact exercises. In fact, BMD is on average higher in athletes with sporting activities involving jumping (volleyball, basketball ball rugby, soccer, and martial arts) compared to those who do not have these mechanical characteristics, such as swimming, rowing, and cycling [36]. Furthermore there are evidences that high level of physical activity during youth, as seen in female athletes, seems to have a beneficial effect on bone mass and helps to prevent bone loss due to aging [37].

In clinical practice, however, the prescription of exercise in the elderly and osteoporotic patient must always be preceded by a careful evaluation: indeed, it is essential to define the type, intensity, and duration of a proposed program. The decision is based on the subject's muscle strength, range of motion, balance, gait, cardiopulmonary function, comorbidities, bone density, and history of previous fractures, as well as the risk of falling [12, 38, 39]. In fact, the most intense exercises, such as high impact activities, that are effective in increasing bone mass in young subjects may not be indicated for some elderly osteoporotic subjects [40]. The progression of the exercise must always be respected, and, in patients with severe osteoporosis, the activities involving the flexion or rotation of the trunk must be avoided.

Regarding aquatic exercise, a recent systematic review supports the evidence of a trend showing its effectiveness in maintaining or even improving BMD [14].

## 2. Materials and Methods 

### 2.1. Search Criteria

Main search engines (PubMed, Cochrane Library, and Pedro) were explored using the keywords: exercise AND osteoporosis in title, resistance exercise AND osteoporosis, weight bearing exercise AND osteoporosis, vibration AND osteoporosis. The following filters were applied: articles in English language on humans aged 65 and over. The research took into account the existing systematic reviews and meta-analyses, focusing also on the individual articles included in the reviews. A subsequent selection regarding only exercise and primary osteoporosis, based on the titles and abstracts analysis of the articles, was performed.

### 2.2. Methodological Problems Emerging from the Studies

Several critical issues in the evaluation of evidence, limiting in some way the conclusion of this review, were highlighted in the Cochrane reviews [3, 15]. Regarding the* methods*, among the several exercises proposed in the literature there were relevant differences in the type and in the setting where the exercises were carried out, the intensity, the duration, frequency of the sessions, and the total duration of the program. Furthermore, the sample size, patients' compliance or adherence to the study, the presence of a control group, the number of postmenopausal years, and the follow-ups reported in the studies were very diverse.

With regard to the* criteria* used to evaluate and measure the effectiveness of the exercises, the studies generally referred to the measurement of BMD detected at the femur (the whole hip, neck, trochanteric and intertrochanteric region, and the Ward's triangle), lumbar spine, distal radius, forearm, tibia, ankle, and total body. In addition to BMD, other studies had also considered aspects such as bone quality, fracture risk reduction, BMC, cortical bone density, body mass, and muscle strength. Although BMD is a relatively good predictor of fracture risk in the elderly population, current research indicates that up to 80% of low-impact trauma fractures occur in individuals who are not osteoporotic but have a normal or slightly reduced BMD, resulting in osteopenia. This discovery highlights the limits of bone densitometry (DXA) in providing accurate BMD measurements or its ability to provide relevant information about the main determinants of bone strength, such as size, shape, and bone structure. Minor changes in bone mass distribution, cortical and trabecular structure, and bone geometry can lead to large increases in bone strength dependent on changes in BMD [15]. The most advanced studies now are focusing on the use of some noninvasive bone imaging techniques, such as quantitative peripheral computed tomography (pQCT), magnetic resonance imaging (MRI), and DXA-based hip structural analysis (HSA) [16, 41].

## 3. Results and Discussion

Forty-four systematic reviews were retrieved in PubMed using the keywords “exercise AND osteoporosis”, 15 using the keywords “resistance exercise AND osteoporosis”, 8 with “weight bearing exercise AND osteoporosis”, and 9 using “vibration AND osteoporosis”. Thirty-three systematic reviews were retrieved from Pedro and 1 Cochrane review from Cochran Library using the keywords “exercise AND osteoporosis”. Of these, once eliminated duplicates and papers not primarily focused on exercise and osteoporosis, 18 systematic reviews and meta-analyses were considered with respect to different type of exercise (Table 1) and 7 with respect to whole body vibration (Table 2).

### 3.1. Weight-Bearing Aerobic Exercise

One of the most common forms of aerobic training is walking, an exercise very well accepted by the older people, because it is harmless, self-managed, and easily practicable. The effects of* walking* on BMD have been widely considered, although the results are not always consistent in the various studies [17–19, 42]. The meta-analyses showed the absence of significant effects on the lumbar spine or on the femoral neck attempted by the only walking [6, 43]. Likewise, from the studies analysed by Gomez Cabello et al. [4], there is no evidence of a close correlation between BMD increasing and gait exercise. However, the effectiveness of walking in maintaining the level of BMD and in preventing its loss is already an excellent result of this simple type of exercise.

Furthermore, it is important to consider some parameters that can influence the effects of walking, such as walking speedily/slowly or strongly/weekly. Actually, there is evidence that an intervention of more than 6 months in duration can provide significant and positive effects on femoral neck BMD in peri- and postmenopausal women [18].

Some studies show how a brisk walking or jogging can have positive effects on hip and column BMD in women of menopausal age [19]. Certainly, some low-impact activities, such as jogging combined with stair climbing and walking, favor minor loss of BMD in both the hip and the spine in menopausal women. Hence, walking/jogging must reach a sufficient high level of mechanical stress determining an important ground reaction force able to stimulate bone mass [9, 10, 20, 41].

In comparing different types of physical exercises with controls, Howe [15] found a significant effect in BMD for bipodalic or monopodalic static exercises and on spine and wrist for dynamic low-impact exercises (including walking and Tai Chi).

Regarding Tai Chi, the issue is in increasing debate; recent literature [21, 22, 44–46] suggests a positive effect on attenuating BMD loss at the lumbar spine and the proximal femoral neck and on biomarkers of bone metabolism. However, in order to be effective, this activity has to last 12 months as minimum [21].

*In summary: *

- *Walking, as an isolated intervention, is not able to modify the loss of BMD. However, in the context of a health maintenance program in general it is advisable to walk for at least 30 minutes a day*.
- *Aerobic training and in particular the path with high intensity and speed, interspersed with jogging, climbing scales, and stepping, is able to limit the reduction of BMD*.

### 3.2. Strength and Resistance Exercises

Strength and resistance training are the most studied techniques to increase bone mass in the elderly. The rationale of these exercises lies in the mechanical stimulus indirectly produced on the bone [8, 9, 41]. Like weight-bearing exercises, the strength exercise determines a joint reaction force and muscle strengthening, producing an important clinical benefit on the BMD, in the lumbar spine and, to a greater extent, in the femoral neck [20]. This type of activity is also defined as “nonimpacting” and can be carried out with loading (lifting weights) or without loading (swimming, cycling) [10].

Studies have examined the effect of strengthening the muscles of the upper limb and lower limb, rather than specific groups such as iliopsoas and spinal extensors. From the evidence gathered by Zehnacker et al. [11] the effectiveness of strength training in the hip and spine sites is related to the intensity of the training; the exercise requires high loads (70-90% of a maximum repetition) for 8-10 repetitions of 2-3 sets performed at least for 1 year, 3 times a week for 45-70 minutes per session. In particular some types of exercise would be able to increase bone mineral density: (1) weighted squats, hack squats, leg press, hip extension, hip adduction, knee extension, and hamstring curls; (2) stair-climbing/step boxes with weighted vests, power cleans with weighted vests, and beverage boxes; (3) military press, latissimus pull down, seated rowing, and rotary torso; (4) back extension exercises with weighted backpack, leg press, bench press, trunk extension, elbow flexion, wrist curl, reverse wrist curl, triceps extension, and forearm pronation and supination.

In relation to the hip, the exercise is effective on the greater trochanter if it involves the buttocks, on the lesser trochanter if it involves the iliopsoas, and on the Ward's triangle if it involves the adductors and the hip extensors, according to the studies of Kerr et al. [47]. Here, the authors concluded that there are several possible explanations for the different effectiveness of site-specific exercises: various muscle insertions, different weight or type of contraction, and duration and nature of the exercise.

Similarly, Sinaki et al. [48] have shown that the strength of the back muscles in osteoporotic women is significantly reduced compared to healthy subjects; therefore, the strengthening of these muscles can reduce the risk of vertebral fractures with simple programs of antigravity extension in the prone position. After two years of exercise, there was a significant reduction in the loss of BMD in the subjects being treated. This significant difference, compared to controls, was maintained eight years after, despite the decrement of both BMD and muscle strength.

In opposite opinion are Bemben et al. [49] who investigated the dose-response effects of resistance training on BMD in elderly women and concluded that the gain in BMD at the proximal femur and lumbar spine is independent of intensity and frequency of isotonic exercise (with Cybex) of the upper and lower limbs. In particular, there was no difference between men and women at the femur level, while in women the effect is greater at the spine. In fact, it seems that the bone in menopausal age can be significantly increased by a regime of strengthening exercises with “high-load low repetitions” but not by a regime of resistance exercises with “low-load high repetitions” [50]. The peak load exerted seems to be therefore more important than the number of repetitions on the increase in bone mass in menopausal women [1]. Other considerations that must be taken into account [9] are that women require a greater intensity of exercise to obtain certain results on bone mass. Hence, it is always important to perform a balanced agonists and antagonists training taking into account that the speed of execution during the movements is pertinent in obtaining greater osteogenic stimulation. The effectiveness of progressive resistance training is confirmed also in the review of Cheung and Giangregorio [41] who considered this exercise the best one in postmenopausal women to improve both spine and hip BMD. This would not be the case of older adults, in which physical activity and exercise only have minimal effects on BMD, while strength training should be suggested. However, in clinical practice, in osteoporotic individuals with high risk of vertebral fracture, the use of resistance machines should be well thought out, since this technique often requires forward bending and twisting of the trunk to perform the exercise or to adjust the equipment and to ensure the proper setting. They can be utilized only if is used and adjusted with the proper form [51].

*In summary*:

- *Strength training determines an increase in specific site bone density, in particular at the neck of the femur and at the lumbar spine, which is maintained in the short to medium term. At least 3 sessions a week for a year are recommended*.
- *Progressive resistance training for the lower limbs is the most effective type of exercise intervention on bone mineral density (BMD) for the neck of femur*.

### 3.3. Multicomponent Training

The multicomponent training consists of a combination of different exercises (aerobics, strengthening, progressive resistance, balancing, and dancing) and it is aimed at increasing or preserving bone mass. This implies that the same interventions are provided to all people, differently from multifactorial training, customized on the individual characteristics [52].

The association of several types of exercise is advised to the patients affected by osteoporosis with the goal to counter the reduction of bone mass [23–26, 43, 53–56]. The combination of multiple types of exercise would have a significant effect on BMD at three sites: femoral neck and greater trochanter, but the maximum benefit would be achieved at the spine level [15].

However, diverse methodological and reporting discrepancies with respect to the proposed mix of exercises, the characteristics of patients with or without fractures, and the outcome measures seem relevant in determining the result of the exercise program. The revision of Gomez-Cabello et al. [1] reports substantially two studies [57, 58] that demonstrate a significant improvement in BMD at the level of the lumbar spine, the neck of the femur, and the greater trochanter, following programs including muscle strengthening and impact exercises. The meta-analysis conducted by Nikander et al. [6] reports in postmenopausal women different results about the effects of the exercise. While resistance training seems to have a good effect on lumbar BMD, the association of this type of exercise with so-called “low-moderate” impact exercises such as jogging, walking, and stair climbing is much more effective in preserving BMD at both lumbar and femoral level [27].

It is interesting to note from this review how the most challenging high impact exercise programs, such as jumping, are only effective when they are associated with other low-impact exercises. Bolan at al. [19] report in their systematic review a positive osteogenic effect of resistance training alone or associated with high impact weight-bearing activities and recall that the intensity and increment of the type of load are two fundamental elements of exercise to avoid adaptation phenomena and produce an improvement on bone mass rather than just decreasing the loss.

Giangregorio et al. [51] stressed the importance for individuals with osteoporosis and osteoporotic vertebral fracture to engage in a multicomponent exercise program with resistance training combined with balance training. In particular, it is stated that such individuals should not engage in aerobic training to the exclusion of resistance or balance training.

Xu et al. [28] quantified the frequency with which a multicomponent training must be carried out in order to be effective. They suggest that each session should be between 30 and 60 min, 3 or more times per week for at least 10 months.

Also, in the review of studies analyzed by Marquez et al. [59, 60], the combination of non-high-impact weight-bearing exercises for muscle strengthening, resistance, aerobic, and balance exercises determined an increase in BMD at the lumbar spine and femoral neck in elderly subjects. According to this group, a multicomponent exercise program with moderate-high impact (marching on the spot, stepping at 120/125 b/m, on a bench of 15 cm, and heel drops on a rigid surface) was able to determine an increase in BMD in the femoral neck in a population of elderly women who had never performed exercise programs before.

*In summary:*

- *Combined exercise and group exercise programs, including weight-bearing activities, balance training, jogging, low-impact loading, high magnitude exercise, muscle strength, and simulated functional tasks, are advised to determine BMD increasing or at least to preserve it. However the combination of exercise should be tailored on the patient's clinical features. No agreement exists on the best protocol in terms of duration, frequency, and the type of exercises to be combined. The most relevant effect was detected at the spine*.

### 3.4. Training with Vibrating Platforms

The vibration of the entire body is a physiotherapy intervention based on the use of a high frequency mechanical stimulus generated by a vibrating platform (Whole Body Vibration or WBV) that activates the mechanoreceptors of the bone favoring osteogenesis. The results of the studies included in two systematic reviews [1, 61] conclude that the treatment with a WBV seems to be more effective than simple walking and of similar efficacy to strength training to improve bone mass at specific sites (femoral neck and spine) in postmenopausal women.

To obtain these results, Dionello et al. [61] report an average duration of the training from 2 to 22 months with one or two weekly sessions lasting from 4 to 20 minutes, with vertical or horizontal vibratory energy, at a variable frequency from 12 Hz to 90 Hz with amplitude from 0.7 mm to 12 mm. The positive effect of WBV in improving BMD in different sites is supported also by other reviews [29–31] and confirmed by Oliveira et al. [32] concluding that, despite WBV presenting potential to act as a coadjutant in the prevention or treatment of osteoporosis, especially for BMD of the lumbar spine, the ideal intervention is not yet clear.

Fratini et al. [33] also claim that whole body vibration produces significant BMD improvements on the hip and spine when compared to no intervention, while treatment associated with exercise training resulted in negligible outcomes when compared to exercise training or to placebo. The authors specify that the most osteogenic effect is obtained with side-alternating platforms, due to the similarity of the stimulus with gait, mechanical oscillations of magnitude higher than 3 g, and/or frequency lower than 25 Hz, while exercising on the platform does not provide further improvement of BMD. Thus, it is better if the subject during the treatment assumes static postures, such as full-standing or hack squat. However, many factors (e.g., amplitude, frequency, and subject posture) affect the capacity of the vibrations to propagate to the target site; the adequate level of stimulation required to produce these effects has not yet been defined.

Conversely, a previous systematic review [34] showed that while the use of vibration platforms can improve muscle strength in the lower limbs of elderly patients, it does not seem to induce significant changes in bone mineral density in women. Similarly, while the analysis performed by Cheung and Giangregorio [41] on 5 systematic reviews shows only a modest clinical improvement of BMD at the hip in postmenopausal women, the review of Jepsen et al. [35] reports only a reduction in fall rate, but not in BMD.

*In summary*:

- *Training with vibrating platforms is reported to have effect on enhancing muscle strength, improving balance, and reducing the risk of fall in osteoporotic patients, while controversial findings on improvement of BMD in different sites were reported*.

## 4. Conclusions 

Although several exercise recommendations for individuals with osteoporosis have been proposed, reviews are often inconclusive, for the methodological variability emerging from the studies.

However, results from the Cochrane review [15] suggest a relatively small, statistically significant, but possibly important effect of exercise on bone density in postmenopausal women compared with control groups. The exercise types most effective on BMD for the neck of femur, which should be considered in clinical practice, appear to be the progressive resistance strength training for the lower limbs. The most effective intervention for BMD at the spine has been suggested to be the multicomponent training exercise programme. Weight-bearing aerobic exercise and training with vibrating platforms may have also an impact in improving BMD. These evidences are relevant not only because they support the possibility to increase BMD in postmenopausal women, but also because they confirm the possibility to prevent further bone loss in osteoporotic patients, thus limiting the risk of fractures. Key considerations for future research lines emerge from this review: (1) the need for studies to evaluate the effects of the longer-term exercise; (2) the need for studies on male patients; (3) the need for studies that use evaluation criteria of the outcome that are more sensitive to changes in the bone structure; (4) inclusion of parameters such as diet or drugs as a covariate in the analysis of the effects of the exercise; (5) the need to stratify studies on the effects of exercise on BMD based on age; and (6) the need to understand the effects of deconditioning.

## Figures and Tables

**Table 1:** Systematic reviews and meta-analyses on Exercise and Osteoporosis.

**Authors/Title/Source**	**Main conclusions**
Gómez-Cabello A, Ara I, González-Agüero A, Casajús JA, Vicente-Rodríguez G. Effects of training on bone mass in older adults: a systematic review. Sports Med. 2012;1;42(4):301-25.[1]	Walking provides a modest increase in the loads on the skeleton above gravity and, therefore, this type of exercise has proved to be less effective in osteoporosis prevention. Strength exercise seems to be a powerful stimulus to improve and maintain bone mass during the ageing process. Multi-component exercise programmes of strength, aerobic, high impact and/or weight-bearing training, as well as whole-body vibration (WBV) alone or in combination with exercise, may help to increase or at least prevent decline in bone mass with ageing, especially in postmenopausal women.

Nikander R, Sievänen H, Heinonen A, Daly RM, Uusi-Rasi K, Kannus P. Targeted exercise against osteoporosis: A systematic review and meta-analysis for optimising bone strength throughout life. BMC Med. 2010 Jul 21;8:47. [6]	Epidemiological evidence suggests that moderate to vigorous physical activity performed three to four times per week is associated with considerably lower incidence of fragility fractures in both women and men. The findings from these studies also suggest that exercise regimens that include moderate- to high-magnitude impacts from varying loading directions may represent the optimal mode to enhance bone structure and strength.

Zehnacker CH, Bemis-Dougherty A. Effect of weighted exercises on bone mineral density in post-menopausal women. A systematic review. J Geriatr Phys Ther. 2007;30(2):79-88. [11]	Weighted exercises can help in maintaining BMD in postmenopausal women and increasing BMD of the spine and hip in women with osteopenia and osteoporosis. The exercise program must be incorporated into a lifestyle change and be lifelong due to the chronic nature of bone loss in older women.

McMahon M. What impact does aquatic therapy have on bone density in postmenopausal women? If it has a positive or maintenance effect, what are the programme parameters that facilitate these outcomes? Aqualines 2017;29(1):8-21.[14]	The majority of the studies reviewed support a trend showing that exercising in water can be useful in at least maintaining, or improving, various measures of bone mineral density (BMD).

Howe T, Shea B, Dawson LJ, Downie F, Murray A, Ross C, Harbour RT, Caldwell LM, Creed G. Exercise for preventing and treating osteoporosis in postmenopausal women. Cochrane Database Syst Rev. 2011 Jul 6;(7):CD000333. [15]	The most effective type of exercise intervention on bone mineral density (BMD) for the neck of femur appears to be non-weight bearing high force exercise such as progressive resistance strength training for the lower limbs. The most effective intervention for BMD at the spine was combination exercise programmes compared with control groups. Our results suggest a relatively small statistically significant, but possibly important, effect of exercise on bone density compared with control groups.

Polidoulis I, Beyene J, Cheung AM. The effect of exercise on pQCT parameters of bone structure and strength in postmenopausal women -- a systematic review and meta-analysis of randomized controlled trials. Osteoporos Int. 2012;23(1):39-51. [16]	We conclude that exercise in postmenopausal women may decrease bone loss by maintaining cortical and trabecular volumetric BMD.

Martyn-St James M1, Carroll S. Meta-analysis of walking for preservation of bone mineral density in postmenopausal women. Plos one. 2008;43(3):521-31. [17]	We conclude that regular walking has no significant effect on preservation of BMD at the spine in postmenopausal women, whilst significant positive effects at femoral neck are evident. However, diverse methodological and reporting discrepancies are apparent in the published trials on which these conclusions are based. Other forms of exercise that provide greater targeted skeletal loading may be required to preserve bone mineral density in this population.

Ma D, Wu L, He Z. Effects of walking on the preservation of bone mineral density in perimenopausal and postmenopausal women: a systematic review and meta-analysis. Menopause. 2013;20(11):1216-26. [18]	Walking as a singular exercise therapy has no significant effects on BMD at the lumbar spine, at the radius, or for the whole body in perimenopausal and postmenopausal women, although significant and positive effects on femoral neck BMD in this population are evident with interventions more than 6 months in duration.

Bolam KA, van Uffelen JG, Taaffe DR. The effect of physical exercise on bone density in middle-aged and older men: a systematic review. Osteop Int. 2013;24(11):2749-62. [19]	Regular resistance training and impact-loading activities should be considered as a strategy to prevent osteoporosis in middle-aged and older men.

Kelley GA, Kelley KS, Kohrt WM. Effects of ground and joint reaction force exercise on lumbar spine and femoral neck bone mineral density in postmenopausal women: a meta-analysis of randomized controlled trials. BMC Musculoskelet Disord. 2012; 20;13:177. [20]	The overall findings suggest that exercise may result in clinically relevant benefits to FN and LS BMD in postmenopausal women.

Chow TH, Lee BY, Ang ABF, Cheung VYK, Ho MMC, Takemura S. The effect of Chinese martial arts Tai Chi Chuan on prevention of osteoporosis: A systematic review. J Orthop Translat. 2017; 26;12:74-84. [21]	TCC is beneficial to BMD and may be a cost-effective and preventive measure of osteoporosis. This beneficial effect is better observed in long-term TCC practice.

Sun Z, Chen H, Berger MR, Zhang L, Guo H, Huang Y. Effects of tai chi exercise on bone health in perimenopausal and postmenopausal women: a systematic review and meta-analysis. Osteoporos Int. 2016 Oct;27(10):2901-11. [22]	Tai chi exercise may have benefits on bone health in perimenopausal and postmenopausal women, but the evidence is sometimes weak, poor, and inconsistent.

de Kam D, Smulders E, Weerdesteyn V, Smits-Engelsman BC. Exercise interventions to reduce fall-related fractures and their risk factors in individuals with low bone density: a systematic review of randomized controlled trials. Osteoporos Int. 2009;20(12):2111-25. [23]	Exercise interventions for patients with osteoporosis should include weight-bearing activities, balance exercise, and strengthening exercises to reduce fall and fracture risk.

Martyn-St James M, Carroll S. A meta-analysis of impact exercise on postmenopausal bone loss: the case for mixed loading exercise programmes. Br J Sports Med. 2009; 43(12):898-908. [24]	Mixed loading exercise programmes combining jogging with other low-impact loading activity and programmes mixing impact activity with high-magnitude exercise as resistance training appear effective in reducing postmenopausal bone loss at the hip and spine. Other forms of impact exercise appear less effective at preserving BMD in this population. However, diverse methodological and reporting discrepancies are evident in current published trials.

Varahra A, Rodrigues IB, MacDermid JC, Bryant D, Birmingham T. Exercise to improve functional outcomes in persons with osteoporosis: a systematic review and meta-analysis. Osteoporos Int. 2018;29(2):265-286. [25]	A multicomponent exercise program of high-speed training combined with simulated functional tasks is promising to enhance functional outcomes. Due to substantial clinical heterogeneity of the target groups and specific demands of exercise modes, it is unclear which exercise program is optimal.

Zhao R, Zhao M, Xu Z. The effects of differing resistance training modes on the preservation of bone mineral density in postmenopausal women: a meta-analysis. Osteoporos Int. 2015; 26(5):1605-18. [26]	Combined resistance exercise protocols appear effective in preserving femoral neck and lumbar spine BMD in postmenopausal women, whereas resistance-alone protocols only produced a nonsignificant positive effect.

Martyn-St James M, Carroll S. Effects of different impact exercise modalities on bone mineral density in premenopausal women: a meta-analysis. J Bone Miner Metab. 2010;28(3):251-67.[27]	Exercise programmes that combine odd- or high-impact activity with high-magnitude resistance training appear effective in augmenting BMD in premenopausal women at the hip and spine. High-impact-alone protocols are effective only on hip BMD in this group. However, diverse methodological and reporting discrepancies are evident in published trials.

Xu J, Lombardi G, Jiao W, Banfi G. Effects of Exercise on Bone Status in Female Subjects, from Young Girls to Postmenopausal Women: An Overview of Systematic Reviews and Meta-Analyses. Sports Med. 2016;46(8):1165-82. [28]	Combined-impact exercise protocols (impact exercise with resistance training) are the best choice to preserve/improve bone mineral density in pre- and postmenopausal women. Whole-body vibration exercises have no beneficial effects on bone in postmenopausal or elderly women.

**Table 2:** Systematic reviews and meta-analyses on Whole Body Vibration.

**Authors/Title/Source**	**Main conclusions**
Slatkovska L, Alibhai SM, Beyene J, Cheung AM. Effect of whole-body vibration on BMD: a systematic review and meta-analysis. Osteoporos Int. 2010;21(12):1969-80.[29]	We found significant but small improvements in BMD in postmenopausal women and children and adolescents, but not in young adults.

Ma C, Liu A, Sun M, Zhu H, Wu H.Effect of whole-body vibration on reduction of bone loss and fall prevention in postmenopausal women: a meta-analysis and systematic review. J Orthop Surg Res. 2016; 17;11:24. [30]	Low-magnitude whole-body vibration therapy can provide a significant improvement in reducing bone loss in the lumbar spine in postmenopausal women.

Merriman H, Jackson K. The effects of whole-body vibration training in aging adults: a systematic review. J Geriatr Phys Ther. 2009;32(3):134-45. [31]	Some but not all of the studies in this review reported similar improvements in muscle performance, balance, and functional mobility with WBV as compared to traditional exercise programs. Bone studies consistently showed that WBV improved bone density in the hip and tibia but not in the lumbar spine.

Oliveira LC, Oliveira RG, Pires-Oliveira DA. Effects of whole body vibration on bone mineral density in postmenopausal women: a systematic review and meta-analysis. Osteoporos Int. 2016;27(10):2913-33. [32]	Despite WBV presenting potential to act as a coadjutant in the prevention or treatment of osteoporosis, especially for BMD of the lumbar spine, the ideal intervention is not yet clear. Our subgroup analyses helped to demonstrate the various factors which appear to influence the effects of WBV on BMD, contributing to clinical practice and the definition of protocols for future interventions.

Fratini A, Bonci T, Bull AM. Whole Body Vibration Treatments in Postmenopausal Women Can Improve Bone Mineral Density: Results of a Stimulus Focussed Meta-Analysis. PLoS One. 2016;11(12):e0166774. [33]	Whole body vibration treatments in elderly women can reduce BMD decline. However, many factors (e.g., amplitude, frequency and subject posture) affect the capacity of the vibrations to propagate to the target site; the adequate level of stimulation required to produce these effects has not yet been defined.

Lau RW, Liao LR, Yu F, Teo T, Chung RC, Pang MY. The effects of whole body vibration therapy on bone mineral density and leg muscle strength in older adults: a systematic review and meta-analysis. Clin Rehab. 2011;25(11):975-88.[34]	Whole body vibration is beneficial for enhancing leg muscle strength among older adults. However, the review suggests that whole body vibration has no overall treatment effect on bone mineral density in older women.

Jepsen DB, Thomsen K, Hansen S, Jørgensen NR, Masud T, Ryg J. Effect of whole-body vibration exercise in preventing falls and fractures: a systematic review and meta-analysis. BMJ Open. 2017; 29;7(12):e018342. [35]	Whole body vibration reduces fall rate but seems to have no overall effect on BMD or microarchitecture.
